# Foot Pain and Disability in Women with Rheumatoid Arthritis, Ehlers–Danlos Syndrome and Systemic Lupus Erythematosus: Relationship with Quality of Life

**DOI:** 10.3390/jcm12196284

**Published:** 2023-09-29

**Authors:** Inmaculada C. Palomo-Toucedo, Gabriel Domínguez-Maldonado, María Reina-Bueno, María del Carmen Vázquez-Bautista, Javier Ramos-Ortega, José Manuel Castillo-López, Pedro V. Munuera-Martínez

**Affiliations:** Department of Podiatry, University of Seville, Calle Avicena, s/n., 41009 Seville, Spain; ipalomo@us.es (I.C.P.-T.); gdominguez@us.es (G.D.-M.); carmenvaz@us.es (M.d.C.V.-B.); jrortega@us.es (J.R.-O.); jmcastillo@us.es (J.M.C.-L.); pmunuera@us.es (P.V.M.-M.)

**Keywords:** rheumatoid arthritis, Ehlers–Danlos syndrome, systemic lupus erythematosus, health-related quality of life, foot pain, foot posture

## Abstract

The aim of this work was to investigate the relationship foot pain and foot disability have with HRQoL in groups of women with RA, SLE and EDS, in comparison with a control group. A cross-sectional study was carried out with females with one of these conditions and a control group. The SF-12 questionnaire was used to collect data about quality of life. The type of foot was classified according to the footprint and the foot posture index. A total of 156 patients and 47 controls participated in the study (*N* = 203). Neither pain nor foot posture were different between groups. The physical and mental components of SF-12 were worse in rheumatoid arthritis and Ehlers–Danlos syndrome patients, and the physical component was worse in systemic lupus erythematosus patients, compared to controls. A significant difference was also observed in the mental component between systemic lupus erythematosus and Ehlers–Danlos syndrome patients, the latter having the lowest values among the groups. We can conclude that women with rheumatoid arthritis, Ehlers–Danlos syndrome, systemic lupus erythematosus and foot pain perceive a worse quality of life. There are no significant changes in foot posture. Pain and health-related quality of life are independent of foot posture.

## 1. Introduction

Rheumatoid arthritis (RA), Ehlers–Danlos syndrome (EDS) and systemic lupus erythematosus (SLE) are rheumatic diseases that predominantly affect women [1,2,3]. Musculoskeletal involvement, especially foot involvement, is one of the main, most prevalent and earliest clinical manifestations, with up to 90% experiencing foot problems during the course of the disease in RA patients [4], up to 77% in the case of SLE patients [5] and up to 81% in EDS patients [6]. Clinical manifestations on the feet of patients with these diseases may be related to a negative impact on health-related quality of life (HRQoL) [7,8,9,10,11,12].

Foot problems may affect the normal gait cycle, alter foot load patterns, inhibit the patient’s movement and, as a consequence, affect physical activity levels and general activities of daily living, which may result in a worse quality of life [13,14]. Pain is one of the most frequent problems in the RA patients’ feet, as well as increased plantar pressure, diminished functional ability, ankle pain or forefoot problems, especially in the metatarsophalangeal joints [15,16,17]. Common deformities include hallux valgus (HV), deformities of the lesser toes due to synovitis and destruction in the metatarsophalangeal joints, and flatfoot [18]. In people with RA, these foot-related problems are strongly associated with a decreased quality of life [4,7,19]. Foot problems are also common manifestations that affect HRQoL in patients with EDS [20,21,22]. Previous research on EDS (hypermobility type) patients’ foot type found that 45% had pes cavus, 27.5% had flatfoot and 27.5% had a normal arch height [23]. Foot pain was also found to be an important complaint among these participants [6,24], as well as other foot alterations, such as, for example, stress concentration in the forefoot that may be related to pain during standing and walking [8]. People with SLE experience a wide range of lower limb and foot manifestations as well [25,26], such as, for example, arthralgia, lesser toe joint deformities, hallux limitus/rigidus, hallux valgus or Tailor’s bunion [12,27]. This musculoskeletal involvement could also be the cause of foot pain in patients with SLE, and may deteriorate the quality of life due to the effect on daily activities, foot function and fear of falling [3,27,28,29,30,31].

A common feature that characterises these rheumatic diseases is that foot involvement may affect HRQoL. However, to the best of the authors’ knowledge, there are no previous studies that compare foot characteristics and their impact on the quality of life in women with AR, EDS and SLE. Therefore, the aim of this work was to investigate the relationship that foot pain and foot disability have with HRQoL in groups of women with RA, SLE and EDS, in comparison with a control group.

## 2. Materials and Methods

This is a cross-sectional study, conducted according to the protocol and good clinical practice principles and Declaration of Helsinki statements. The participants were recruited consecutively between January 2016 and July 2022, and gave their informed consent to be included. The study was approved by the Bioethics Committee of the Government of Andalusia and authorised by the Head Office of the Clinical Area of Podiatry of the University of Seville.

For participant recruitment, the rheumatology units of the hospitals Virgen de Valme, Virgen del Rocio and Virgen Macarena in Seville were contacted, as well as several associations of patients (the Andalusian Rheumatologic League, the Provincial Spondylitis and Arthritis Association, the Sevillian Association of Patients with Rheumatoid Arthritis, the Joint Hypermobility, Collagenopathies and Ehlers–Danlos Syndromes National Association, and the Spanish Lupus Federation). A comparison group was obtained from volunteers who did not suffer from any rheumatic disease.

As most patients in these three rheumatic diseases are female, the current study included only women. The inclusion criteria were adults with a diagnosis of RA according to the criteria of the American Rheumatism Association [29], or with EDS hypermobile type diagnosed by a medical specialist on the basis of the Villefranche criteria [30], or with a diagnosis of SLE confirmed by a consultant rheumatologist and fulfilling the American College of Rheumatology criteria [31]. They also needed to have foot involvement, and no foot orthoses treatment for 30 days prior to the study. Control participants with foot pain but without rheumatic diseases were also included. Patients were excluded if they had rheumatoid arthritis with acute symptomatic flare, ulcers or wounds in their feet, had cutaneous lupus without systemic involvement, had other systemic diseases, such as diabetes mellitus, neurological impairment or cognitive deterioration, were pregnant, had had previous foot surgery or needed walking assistance.

Clinical and demographic data were collected, including age, weight, height, years since diagnosis of the rheumatic disease, foot pain, foot functionality, foot-related disability and quality of life.

Patients were examined and the foot posture index (FPI) (a validated method for quantifying standing foot posture) [32] was used. In addition, any type of toe deformity was recorded (hallux valgus, claw toes, hammer toes or any other deviation in the sagittal, frontal and/or transverse planes).

Pain was measured using the 11-point Numeric Range Scale (11-NRS) [33]. This ranges from 0 (no pain) to 10 (unbearable pain). Pain days were also recorded as the number of days on which the patient felt foot pain in the previous week by assigning a whole number between 0 and 7.

Foot functionality was measured using the foot function index (FFI) [34]. This is a questionnaire with 23 items that are divided into three domains: foot pain, disability and functional limitation. The values range between 0 and 100, with higher values corresponding to greater pain, disability and limitation.

Disability related to foot pain was measured using the Manchester foot pain and disability index (MFPDI) [35]. The values range between 0 and 100, with higher values corresponding to greater disability.

The SF-12 questionnaire was used to collect data about the quality of life [36], which has values between 0 and 100, with higher values corresponding to lower quality of life.

Footprints were obtained from all participants. AutoCad^®^ software 34 version (AutoCAD 2019; Autodesk Inc., San Rafael, CA, USA) was used to calculate the arch index [37]. This index was obtained through the proportion between the area of the central part of the footprint (B) and its total area (A) + (B) + (C). Results less than 0.21 correspond to high arched feet, between 0.21 and 0.26 suggest normal feet, and higher than 0.26 correspond to low arched feet [38].

The minimum sample size was calculated using the variables SF-12 Physical and Mental as a reference, with the following formula used to compare mean values between populations:

$$n=\frac{2s^{2}{\left(z_{\frac{\alpha }{2}}+z_{\beta }\right)}^{2}}{d^{2}}$$

where *s*^2^ is the sample variance based on a previous study on Spanish people in which SF-12 was measured [39], *α* is the type I error, *β* is the type II error and *d* is the minimum difference to be detected. Therefore, the following result was obtained:

$$n=\frac{2s^{2}{\left(z_{\frac{\alpha }{2}}+z_{\beta }\right)}^{2}}{d^{2}}=\frac{2\cdot 8.99^{2}\cdot {\left(1.96+0.84\right)}^{2}}{5.5^{2}}=41.89≅42$$

Thus, at least 42 women were needed in each group to compare the mean values. In this study, 56 women with RA, 48 with EDS, 52 with SLE and 47 controls were recruited.

The analysis of the data was carried out using the statistical software IBM SPSS Statistics 25 version (IBM, Armonk, NY, USA). The descriptive data provided the mean values and the standard deviations or the absolute frequencies and percentages depending on whether the variables were scalar or categorical.

The association among qualitative variables was evaluated using the chi-square test. Corrected typified residuals were used to know which relationship between variable categories was significant in the different groups.

Normality tests were conducted for the inferential analysis to determine the most appropriate test to use. An inferential analysis using the one-way ANOVA or Kruskal–Wallis statistical test was performed. When significant differences were found, multiple comparisons were made using post hoc tests (Bonferroni when homoscedasticity was confirmed by means of the Levene test, or Hotelling’s T2 when not, in the case of ANOVA). A multinomial logistic regression model was then carried out including only the variables whose *p*-values were lower than 0.05. As there were more than 2 categories, the control group was chosen as the reference variable. To evaluate the goodness-of-fit of the logistic model, the pseudo-R-squared (Nagelkerke’s R^2^) was provided. This pseudo-R-squared looks the same as R-squared as it is on a similar scale, ranging from 0 to 1 with higher values indicating a better model fit. The confidence level a priori was 95%.

## 3. Results

One hundred and fifty-six female patients and 47 controls participated in the study (N = 203). Fifty-six of them were RA patients (mean age 59.6 ± 12.0 years, BMI 26.6 ± 5.0 kg/m^2^ and mean time from diagnosis 14.8 ± 12.8 years), 48 had a diagnosis of EDS (mean age 41.7 ± 11.0 years, BMI 23.5 ± 4.7 kg/m^2^ and mean time from diagnosis 2.2 ± 1.8 years) and 52 suffered from SLE (mean age 47.1 ± 11.3 years, BMI 28.0 ± 6.6 kg/m^2^ and mean time from diagnosis 15.7 ± 10.0 years). Forty-seven women formed the control group (mean age 45.5 ± 17.8 years, BMI 26.5 ± 5.7 kg/m^2^). The descriptive data of the study variables are summarized in Table 1.

Table 2 shows the results of comparisons of the quantitative and categorical variables between the groups. Significant differences were obtained in some of the demographic variables, such as age and BMI, between groups, with the women with RA tending to be older than those in the other groups, and the women with EDS tending to have lower BMI than RA and SLE participants. Women with EDS showed fewer years with the disease than those with AR or SLE. Note that neither pain measured with the 11-NRS nor foot posture were different between groups. However, according to the arch index, participants with RA showed more low arched feet than the other participants. The physical and mental components of SF-12 were worse in RA and EDS patients, and the physical component was worse in SLE patients, compared to controls. A significant difference was also observed in the mental component between SLE and EDS patients, the latter having the lowest values among the groups.

The association among foot pain location and toe deformities between groups was evaluated using the chi-square test. The percentage of women with forefoot pain was not different between the disease groups, rearfoot pain was more frequent in EDS patients (*p* = 0.017) and ankle pain and toe deformities were more frequent in participants with RA (*p* = 0.003 and *p* < 0.001, respectively).

The results of the multinomial logistic regression model with the variables that show statistical differences between groups are shown in Table 3. The control group was established as the reference category. General variables related to the quality of life in women with RA were age and BMI, in women with EDS only BMI, and there were none in women with SLE. Foot variables associated with the quality of life were ankle pain in participants with RA and EDS, and none in those with SLE. Nagelkerke’s pseudo R-squared value was 0.870.

## 4. Discussion

The main objective of this work was to study which of the main clinical manifestations observed in the feet of women with RA, EDS or SLE were associated with HRQoL, as foot involvement is frequent in these rheumatic diseases. To the best of the authors’ knowledge, this is the first study that compares foot characteristics and their impact on quality of life in female patients with AR, EDS and SLE.

Previous studies have suggested that foot problems are associated with impairment of HRQoL in patients with rheumatic diseases. With even mild expression of the disease process in their feet, this negative impact in quality of life is worsened [40]. Pain from foot joint movement and restriction has previously been associated with disease activity and disability in people with RA [41]. This may be related to pain in the ankle joint, the main foot variable that correlated with worse quality of life in women with RA and EDS of the present study, both the physical and mental components, compared to controls. Reinoso-Cobo et al. [42] reported similar results, as patients in their study revealed that a higher score on pain, female gender and older age were related to reduced quality of life (physical component).

Foot problems may also deteriorate quality of life and daily activities in patients diagnosed with SLE [10]. Specific foot problems such as, for example, anomalous plantar pressure patterns, are involved in foot discomfort and pain that limit the willingness of people with EDS to maintain their standing posture or walk for prolonged times, which significantly affects their quality of life [8]. EDS patients had significantly higher levels of pain severity that demonstrated a more important impact of pain on daily life for EDS patients compared to RA patients [43]. However, the results of our study revealed similar scores of foot pain and related function and disability between these two diseases, although the physical component of quality of life was worse in EDS participants. The complexity of this syndrome, the overall worse understanding of the disease and the fact that diagnosis of EDS is based upon clinical criteria alone without the support of biochemical studies, which may be the cause of delayed diagnosis or misdiagnosis, could contribute to a poorer functional status and HRQoL in EDS patients compared to RA or SLE [43].

The main clinical manifestations observed in the three groups of participants included in this study were foot pain and foot disability in comparison with the control group. Although foot pain was similar between disease groups and controls when measured with 11-NRS or days of pain (foot pain was an inclusion criterion for control participants), the women in all disease groups showed more foot pain and disability than controls according to the results of the FFI and MFPDI total scores. Previous studies reported similar results [3,27,44].

FFI has been widely used in rheumatology studies, as it has previously been recommended for treatment assessment in rheumatology [45]. Our participants also had higher (worse) FFI values (pain, disability or total scores) than those reported by other authors [46,47,48], and revealed significant differences when comparing to controls, as also observed in previous studies [49]. Differences observed in FFI and MFPDI in our participants compared to other studies might be due to the fact that these studies always included both male and female patients, whereas in the present work, only women participated, although authors who separated data from men and women also reported a lower punctuation of FFI scores in women (better foot function and less pain) [42]. RA participants in Rome et al.’s study (68% female) [50] showed lower FFI Pain scores, and similar FFI Disability scores than the women from the present study. However, MFPDI scores are similar to those reported by other authors [42]. Studies where participants were predominantly women with EDS suggest that daily life activities are strongly restricted by the foot pain and related disability, as reported by the MFPDI [21].

Although women with rheumatic diseases show significant differences in the punctuation of FFI and MFPDI compared to controls, the foot-related variables that best correlate with a poorer quality of life are the presence of pain in the ankle for RA and EDS women, or in the forefoot in SLE patients. Forefoot pain in SLE may be the consequence of severe local joint inflammation presenting in metatarsophalangeal joints [24,28]. The arch index has suggested that RA patients have lower longitudinal arches than EDS or SLE patients, which is a foot characteristic widely reported in these patients [51,52].

However, the results obtained from the present study show that foot posture does not differ significantly in either group. For this reason, this variable should not be considered as the cause of poor HRQoL in the present sample. Some authors sustain that FPI shows high values (pronated feet) in patients with RA [53], EDS [21] and SLE [54]. However, in the authors’ opinion, foot posture may be altered after foot pain appearance, especially when these autoimmune diseases are not well controlled. This is supported by the results of Bal et al. [55] and Göksel Karatepe et al. [56], who observed that the participants in the control group showed more prevalence of flat feet than the RA group. Reinoso-Cobo et al. [42] affirmed that morphological and structural characteristics of the foot were not necessarily associated with pain, disability and loss of function in RA patients. In contrast, the study by Gonzalez-Fernandez [53] reported a higher percentage of flattened footprint and pronated FPI in people with RA compared to the control group, although they also highlighted that they found more supinated feet in the RA group than in the control group. Biscontini et al. [57] reported 65.4% of pronated feet, suggesting this foot position is more frequent in patients with AR. This disagrees with the data obtained in this study, where FPI values corresponded to a normal foot. Our participants with RA showed lower arch height in their feet than those in the other groups, maybe because the RA women were older and had more time since diagnosis. According to these results, RA, EDS and SLE are related to a high level of foot pain that may negatively influence the quality of life of these patients. Control of these symptoms could have a positive effect on quality of life. Bearing in mind that the participants also presented very similar foot postures, we can deduce that biomechanical alterations and alterations in the plantar arch may have a lesser influence on the appearance of pain and, indirectly, on quality of life. From a clinical point of view, the prevention of deformities through foot orthoses that avoid metatarsophalangeal deviations, redistribute plantar pressure, cushion the areas with high pressure and stabilize the foot could significantly improve the quality of life of these patients [48].

This study has certain limitations. Firstly, the potential impact of pain medication on HRQoL was not considered. However, we think that even with the use of pain alleviating medication, pain levels were similar between the four groups and only the presence of pain in different areas of the foot was significantly related with impaired HRQoL. The evolution time of pain can affect HRQoL, but this item is not included in the questionnaires and scales used in this research, so it was not analysed. In addition, footwear was not considered. The use of inappropriate shoes [58] is very common in young women and may have a negative influence when the rheumatic disease is not established yet, as foot involvement occurs in early disease. This factor should be studied as well as its effect on the increase in foot problems and HRQoL in rheumatic patients.

## 5. Conclusions

According to the results shown, we can conclude that women with RA, EDS, SLE and foot pain perceive worse HRQoL. Metatarsophalangeal deformities are more prevalent in rheumatic diseases; however, there are no significant changes in foot posture. Pain and HRQoL are independent of foot posture.

## Figures and Tables

**Table 1:** Sample descriptive data.

	RA		EDS		SLE		Control	
	N = 56 (27.6%)		N = 48 (23.6%)		N = 52 (25.6%)		N = 47 (23.2%)	
	Mean	SD	Mean	SD	Mean	SD	Mean	SD
Age	59.6	12.0	41.7	11.0	47.1	11.3	45.5	17.8
BMI	26.6	5.0	23.5	4.7	28.0	6.6	26.5	5.7
Years since diagnosis	14.8	12.8	2.2	1.8	15.7	10.0		
VAS	6.5	2.4	6.2	2.1	6.6	1.9	5.7	2.6
FPI right foot	5.0	4.2	4.7	2.8	4.1	3.5	4.0	4.0
FPI left foot	5.1	4.1	4.6	2.8	4.2	3.4	4.1	3.8
Days of pain	5.8	1.9	5.7	2.2	5.1	2.3	5.4	5.3
FFI Pain	63.7	21.9	67.4	19.4	65.5	18.0	48.4	25.7
FFI Disability	53.5	29.4	51.7	26.0	51.4	26.7	27.9	28.4
FFI Functional limitation	19.7	20.2	24.4	25.6	17.2	19.4	12.4	14.0
FFI Total	50.0	20.7	52.0	19.9	49.1	20.0	32.2	21.4
MFPDI Functional	12.1	4.9	13.2	5.1	11.4	5.3	7.3	5.5
MFPDI Appearance	1.4	1.5	0.6	0.9	0.9	1.4	0.5	1
MFPDI Pain	6.8	2.4	6.0	2.0	6.5	2.2	4.6	2.8
MFPDI Work	2.5	1.6	2.6	1.5	2.0	1.7	1.0	1.4
MFPDI Total	22.4	8.2	22.5	7.8	20.8	8.6	13.3	9.1
SF12 Physical	31.9	9.5	26.5	9.2	30.7	8.8	45.1	9.9
SF12 Mental	36.0	11.2	34.9	11.8	43.3	11.7	48.3	9.4
Arch index right foot	0.27	0.04	0.22	0.06	0.23	0.05	0.23	0.09
Arch index left foot	0.27	0.04	0.23	0.05	0.23	0.07	0.25	0.09
Pain location in the foot	N	%	N	%	N	%	N	%
Forefoot	43	76.8	29	60.4	40	76.9	25	53.2
Rearfoot	18	32.1	20	41.7	14	26.9	6	12.8
Ankle	22	39.3	17	35.4	14	26.9	4	8.5
Hallux valgus	36	65	21	44.7	27	51.9	22	48.9
Toe deformities	49	87.5	28	59.6	31	59.6	18	41.9

RA: rheumatoid arthritis; EDS: Ehlers–Danlos syndrome; SLE: systemic lupus erythematosus; BMI: body mass index; VAS: visual analogue scale; FPI: foot posture index; FFI: foot function index; MFPDI: Manchester foot pain and disability index.

**Table 2:** Variable comparisons between groups.

	*p*	RA-EDS	RA-SLE	RA-C	EDS-SLE	EDS-C	SLE-C
Age	<0.001	<0.001	<0.001	<0.001			
BMI	0.001	0.009		N/A	0.001	N/A	N/A
Years since diagnosis	<0.001	<0.001		N/A	<0.001	N/A	N/A
VAS	0.436						
FPI right foot	0.181						
FPI left foot	0.301						
Days of pain	0.133						
FFI Pain	0.001			0.013		0.001	0.005
FFI Disability	<0.001			<0.001		0.001	<0.001
FFI Functional limitation	0.082						
FFI Total	<0.001			<0.001		<0.001	0.001
MFPDI Functional	<0.001			<0.001		<0.001	0.004
MFPDI Appearance	0.003	0.031		0.003			
MFPDI Pain	<0.001			<0.001			0.004
MFPDI Work	<0.001			<0.001		<0.001	0.010
MFPDI Total	<0.001			<0.001		<0.001	0.002
SF12 Physical	<0.001	0.014		<0.001		<0.001	<0.001
SF12 Mental	<0.001		0.018	<0.001	0.004	<0.001	
Arch index right foot	<0.001	0.001	0.001	0.001			
Arch index left foot	0.002	0.006	0.023	0.010			

RA: rheumatoid arthritis; EDS: Ehlers–Danlos Syndrome; SLE: systemic lupus erythematosus; BMI: body mass index; VAS: visual analogue scale; FPI: foot posture index; FFI: foot function index; MFPDI: Manchester foot pain and disability index. N/A: non-applicable.

**Table 3:** Multinomial logistic regression data.

Rheumatoid Arthritis	B	SE	P	Exp(B)	Exp(B) 95% CI	
Coefficient (intercept)	30.147	8.617	0.000			
Age	0.160	0.049	0.001	1.174	1.065	1.293
BMI	−0.296	0.107	0.006	0.744	0.603	0.917
FFI Pain	0.056	0.046	0.232	1.057	0.965	1.158
FFI Disability	0.027	0.054	0.613	1.028	0.925	1.142
FFI Total	−0.135	0.098	0.167	0.874	0.721	1.058
MFPDI Functional	−0.601	0.423	0.155	0.548	0.239	1.256
MFPDI Appearance	0.341	0.486	0.483	1.407	0.542	3.651
MFPDI Pain	−0.181	0.534	0.735	0.835	0.293	2.376
MFPDI Work	−0.512	0.674	0.447	0.599	0.160	2.244
MFPDI Total	0.388	0.408	0.340	1.475	0.664	3.278
SF12 Physical	−0.382	0.099	0.000	0.682	0.562	0.829
SF12 Mental	−0.197	0.056	0.000	0.821	0.736	0.915
Forefoot pain	−0.846	1.100	0.442	0.429	0.050	3.710
Rearfoot pain	1.551	1.216	0.202	4.714	0.435	51.121
Ankle pain	−4.115	1.448	0.004	0.016	0.001	0.279
Toe deformities	−3.834	1.206	0.001	0.022	0.002	0.230
Arch index right foot						
High	−1.606	1.905	0.399	0.201	0.005	8.401
Low	1.640	1.375	0.233	5.156	0.348	76.350
Arch index left foot						
High	0.502	1.608	0.755	1.652	0.071	38.638
Low	1.070	1.412	0.449	2.914	0.183	46.404
**Ehlers–Danlos syndrome**	**B**	**SE**	**P**	**Exp(B)**	**Exp(B) 95% CI**	
Coefficient (intercept)	48.562	9.506	0.000			
Age	−0.024	0.048	0.618	0.976	0.889	1.073
BMI	−0.414	0.121	0.001	0.661	0.521	0.839
FFI Pain	0.098	0.052	0.058	10.103	0.996	1.222
FFI Disability	0.004	0.051	0.942	1.004	0.909	1.109
FFI Total	−0.059	0.096	0.538	0.942	0.780	1.138
MFPDI Functional	−0.221	0.522	0.672	0.802	0.288	2.231
MFPDI Appearance	−0.707	0.664	0.287	0.493	0.134	1.811
MFPDI Pain	−0.269	0.633	0.671	0.764	0.221	2.643
MFPDI Work	−0.736	0.837	0.379	0.479	0.093	2.472
MFPDI Total	−0.002	0.507	0.997	0.998	0.370	2.693
SF12 Physical	−0.506	0.103	0.000	0.603	0.492	0.738
SF12 Mental	−0.297	0.064	0.000	0.743	0.655	0.843
Forefoot pain	1.445	1.208	0.232	4.241	0.397	45.264
Rearfoot pain	−0.422	1.271	0.740	0.656	0.054	7.919
Ankle pain	−3.004	1.494	0.044	0.050	0.003	0.927
Toe deformities	−2.065	1.146	0.071	0.127	0.013	1.197
Arch index right foot						
High	1.025	1.431	0.474	2.788	0.169	46.081
Low	0.797	1.680	0.635	2.219	0.082	59.766
Arch index leftfoot						
High	0.271	1.558	0.862	1.312	0.062	27.821
Low	−0.093	1.735	0.957	0.911	0.030	27.284
**Systemic lupus erythematosus**	**B**	**SE**	**P**	**Exp(B)**	**Exp(B) 95% CI**	
Coefficient (intercept)	23.985	7.605	0.002			
Age	0.035	0.033	0.293	1.035	0.971	1.104
BMI	−0.103	0.079	0.191	0.902	0.773	1.053
FFI Pain	0.041	0.031	0.187	10.042	0.981	1.106
FFI Disability	−0.014	0.040	0.729	0.986	0.912	1.067
FFI Total	−0.008	0.059	0.892	0.992	0.883	1.114
MFPDI Functional	0.027	0.398	0.945	1.028	0.471	2.244
MFPDI Appearance	0.005	0.457	0.992	1.005	0.410	2.462
MFPDI Pain	0.076	0.507	0.880	1.079	0.399	2.917
MFPDI Work	−0.864	0.654	0.186	0.422	0.117	1.518
MFPDI Total	−0.108	0.390	0.783	0.898	0.418	1.928
SF12 Physical	−0.350	0.091	0.000	0.704	0.590	0.842
SF12 Mental	−0.098	0.046	0.034	0.906	0.828	0.993
Forefoot pain	−1.273	0.914	0.164	0.280	0.047	1.680
Rearfoot pain	0.631	1.030	0.540	1.880	0.250	14.153
Ankle pain	−2.039	1.318	0.122	0.130	0.010	1.723
Toes deformities	−0.720	0.832	0.386	0.487	0.095	2.484
Arch index right foot						
High	0.560	1.096	0.610	1.751	0.204	15.012
Low	−0.795	1.300	0.541	0.452	0.035	5.771
Arch index left foot						
High	0.778	1.266	0.539	2.177	0.182	26.047
Low	1.366	1.363	0.316	3.920	0.271	56.651

SE: standard error. BMI: body mass index; FFI: foot function index; MFPDI: Manchester foot pain and disability index. For arch index, “normal arch” category was the reference variable.

## Data Availability

Not applicable.

## References

[1] Vermeulen S., De Mits S., De Ridder R., Calders P., De Schepper J., Malfait F., Rombaut L. (2022). Altered Multisegment Ankle and Foot Kinematics During Gait in Patients With Hypermobile Ehlers-Danlos Syndrome/Hypermobility Spectrum Disorder: A Case-Control Study. Arthritis Care Res..

[2] Moreira E., Jones A., Oliveira H., Jennings F., Fernandes A., Natour J. (2016). Effectiveness of insole use in rheumatoid feet: A randomized controlled trial. Scand. J. Rheumatol..

[3] Cherry L., Alcacer-Pitarch B., Hopkinson N., Teh L.S., Vital E.M., Edwards C.J., Blake A., Williams A.E., Alves E.M., Macieira J.C. (2017). The prevalence of self-reported lower limb and foot health problems experienced by participants with systemic lupus erythematosus: Results of a UK national survey. Lupus.

[4] Wilson O., Hewlett S., Woodburn J., Pollock J., Kirwan J. (2017). Prevalence, impact and care of foot problems in people with rheumatoid arthritis: Results from a United Kingdom based cross-sectional survey. J. Foot Ankle Res..

[5] Otter S.J., Kumar S., Gow P., Dalbeth N., Corkill M., Rohan M., Davies K.A., Pankathelam S., Rome K. (2016). Patterns of foot complaints in systemic lupus erythematosus: A cross sectional survey. J. Foot Ankle Res..

[6] Reina-Bueno M., Vázquez-Bautista C., Palomo-Toucedo I.C., Domínguez-Maldonado G., Castillo-López J.M., Munuera-Martínez P.V. (2020). Custom-Made Foot Orthoses Reduce Pain and Fatigue in Patients with Ehlers-Danlos Syndrome. A Pilot Study. Int. J. Environ. Res. Public Health.

[7] De Souza S., Lempp H. (2017). PARE0010 Significance of foot problems for patients with rheumatoid arthritis: A patient-led qualitative study. Ann. Reum. Dis..

[8] Pau M., Galli M., Celletti C., Morico G., Leban B., Albertini G., Camerota F. (2013). Research in Developmental Disabilities Plantar pressure patterns in women affected by Ehlers—Danlos syndrome while standing and walking. Res. Dev. Disabil..

[9] Castori M. (2012). Ehlers-Danlos Syndrome, Hypermobility Type: An Underdiagnosed Hereditary Connective Tissue Disorder with Mucocutaneous, Articular, and Systemic Manifestations. ISRN Dermatol..

[10] Williams A.E., Blake A., Cherry L., Alcacer-Pitarch B., Edwards C.J., Hopkinson N., Vital E.M.J., Teh L.S. (2017). Patients’ experiences of lupus-related foot problems: A qualitative investigation. Lupus.

[11] McElhone K., Abbott J., Teh L.S. (2006). A review of health related quality of life in systemic lupus erythematosus. Lupus.

[12] Stevens M.J., Walker-Bone K., Culliford D.J., Alcacer-Pitarch B., Blake A., Hopkinson N., Teh L.S., Vital E.M., Edwards C.J., Williams A.E. (2019). Work participation, mobility and foot symptoms in people with systemic lupus erythematosus: Findings of a UK national survey. J. Foot Ankle Res..

[13] Santos D. (2015). The Effects of off-the-Shelf Foot Orthoses on the Quality of Life of Patients Diagnosed with Early Rheumatoid Arthritis. Clin. Res. Foot Ankle.

[14] Tenten-diepenmaat M. (2018). The effectiveness of therapeutic shoes in patients with rheumatoid arthritis: A systematic review and meta-analysis. Rheumatol. Int..

[15] Novak P., Burger H., Tomsic M., Marincek C., Vidmar G. (2009). Influence of foot orthoses on plantar pressures, foot pain and walking ability of rheumatoid arthritis patients—A randomised controlled study. Disabil. Rehabil..

[16] Stolt M., Kilkki M., Katajisto J., Suhonen R. (2021). Self-assessed foot health in older people with rheumatoid arthritis—A cross-sectional study. Int. J. Older People Nurs..

[17] Perisano C., Cannella A., Polichetti C., Mascio A., Comisi C., De Santis V., Caravelli S., Mosca M., Spedicato G.A., Maccauro G. (2023). Tibiotalar and Tibiotalocalcaneal Arthrodesis with Paragon28 SilverbackTM Plating System in Patients with Severe Ankle and Hindfoot Deformity. Medicina.

[18] Mochizuki T., Yano K., Ikari K., Hiroshima R., Ishibashi M., Okazaki K. (2020). Relationship of callosities of the forefoot with foot deformity, Health Assessment Questionnaire Disability Index, and joint damage score in patients with rheumatoid arthritis. Mod. Rheumatol..

[19] Santos D., Cameron-Fiddes V. (2014). Effects of Off-the-Shelf Foot Orthoses on Plantar Foot Pressures in Patients with Early Rheumatoid Arthritis. J. Am. Podiatr. Med. Assoc..

[20] Voermans N.C., Knoop H., Bleijenberg G., Van Engelen B.G. (2010). Pain in Ehlers-Danlos Syndrome is common, severe, and associated with functional impairment. J. Pain Symptom Manag..

[21] Berglund B., Nordström G., Hagberg C., Mattiasson A. (2005). Foot pain and disability in individuals with Ehlers-Danlos syndrome (EDS): Impact on daily life activities. Disabil. Rehabil..

[22] Rombaut L., Malfait F., Cools A., De Paepe A., Calders P. (2010). Musculoskeletal complaints, physical activity and health-related quality of life among patients with the EhlersDanlos syndrome hypermobility type. Disabil. Rehabil..

[23] Cimolin V., Galli M., Celletti C., Pau M., Castori M., Morico G., Albertini G., Camerota F. (2014). Foot type analysis based on electronic pedobarography data in individuals with joint hypermobility syndrome/Ehlers-Danlos syndrome hypermobility type during upright standing. J. Am. Podiatr. Med. Assoc..

[24] Berglund B., Nordström G., Lützén K. (2000). Living a restricted life with Ehlers-Danlos Syndrome (EDS). Int. J. Nurs. Stud..

[25] Iagnocco A., Ceccarelli F., Rizzo C., Truglia S., Massaro L., Spinelli F.R., Vavala C., Valesini G., Conti F. (2014). Ultrasound evaluation of hand, wrist and foot joint synovitis in systemic lupus erythematosus. Rheumatology.

[26] Stewart S., Brenton-Rule A., Dalbeth N., Aiyer A., Frampton C., Rome K. (2019). Foot and ankle characteristics in systemic lupus erythematosus: A systematic review and meta-analysis. Semin. Arthritis Rheum..

[27] Morales-Lozano R., Martínez-Barrio J., González-Fernández M.L., López-Longo F.J., Ovalles-Bonilla J.G., Valor L., Janta I., Nieto J.C., Hernández-Flórez D., González C.M. (2016). The feet in systemic lupus erythematosus; are we underestimating their involvement and functional impact?. Clin. Exp. Rheumatol..

[28] Mukherjee S., Cherry L., Zarroug J., Culliford D., Bowen C., Arden N., Edwards C. (2016). A pilot investigation of the prevalence of US-detectable forefoot joint pathology and reported foot-related disability in participants with systemic lupus erythematosus. J. Foot Ankle Res..

[29] Arnett F., Edworthy S., Bloch D., McShane D., Fries J., Cooper N., Healey L., Kaplan S., Liang M., Luthra H. (1988). The American Rheumatism Association 1987 revised criteria for the classification of rheumatoid arthritis. Arthritis Rheum..

[30] Beighton P., De Paepe A., Steinmann B., Tsipouras P., Wenstrup R.J. (1998). Ehlers-Danlos syndromes: Revised nosology, Villefranche, 1997. Ehlers-Danlos National Foundation (USA) and Ehlers-Danlos Support Group (UK). Am. J. Med. Genet..

[31] Hochberg M.C. (1997). Updating the American College of Rheumatology revised criteria for the classification of systemic lupus erythematosus. Arthritis Rheum..

[32] Redmond A.C., Crosbie J., Ouvrier R.A. (2006). Development and validation of a novel rating system for scoring standing foot posture: The Foot Posture Index. Clin. Biomech..

[33] Landorf K.B., Radford J.A. (2008). Minimal important difference: Values for the Foot Health Status Questionnaire, Foot Function Index and Visual Analogue Scale. Foot.

[34] Paez-Moguer J., Budiman-Mak E., Cuesta-Vargas A.I. (2014). Cross-cultural adaptation and validation of the Foot Function Index to Spanish. Foot Ankle Surg..

[35] Gijon-Nogueron G., Ndosi M., Luque-Suarez A., Alcacer-Pitarch B., Munuera P.V., Garrow A., Redmond A.C. (2014). Cross-cultural adaptation and validation of the Manchester Foot Pain and Disability Index into Spanish. Qual. Life Res..

[36] Vera-Villarroel P., Silva J., Celis-Atenas K., Pavez P. (2014). Evaluación del cuestionario SF-12: Verificación de la utilidad de la escala de salud mental. Rev. Med. Chil..

[37] Reina M., Lafuente G., Munuera P.V. (2013). Effect of custom-made foot orthoses in female hallux valgus after one-year follow up. Prosthet. Orthot. Int..

[38] Cavanagh P.R., Rodgers M.M. (1987). The arch index: A useful measure from footprints. J. Biomech..

[39] Rodríguez Moreno I., Ballesteros-Mora M., Reina-Bueno M. (2017). Relación de la calidad de vida y los autocuidados podológicos con las complicaciones asociadas a la diabetes. Estudio descriptivo. Rev. Española Podol..

[40] Wickman A.M., Pinzur M.S., Kadanoff R., Juknelis D. (2004). Health-Related Quality of Life for Patients with Rheumatoid Arthritis Foot Involvement. Foot Ankle Int..

[41] Rojas-Villarraga A., Bayona J., Zuluaga N., Mejia S., Hincapie M.E., Anaya J.M. (2009). The impact of rheumatoid foot on disability in Colombian patients with rheumatoid arthritis. BMC Musculoskelet. Disord..

[42] Reinoso-Cobo A., Gijon-Nogueron G., Caliz-Caliz R., Ferrer-Gonzalez M.A., Vallejo-Velazquez M.T., Miguel Morales-Asencio J., Ortega-Avila A.B. (2020). Foot health and quality of life in patients with rheumatoid arthritis: A cross-sectional study. BMJ Open.

[43] Rombaut L., Malfait F., De Paepe A., Rimbaut S., Verbruggen G., De Wandele I., Calders P. (2011). Impairment and impact of pain in female patients with Ehlers-Danlos syndrome: A comparative study with fibromyalgia and rheumatoid arthritis. Arthritis Rheum..

[44] Otter S.J., Lucas K., Springett K., Moore A., Davies K., Young A., Walker-Bone K. (2011). Comparison of foot pain and foot care among rheumatoid arthritis patients taking and not taking anti-TNFalpha therapy: An epidemiological study. Rheumatol. Int..

[45] Budiman-Mak E., Conrad K.J., Mazza J., Stuck R.M. (2013). A review of the foot function index and the foot function index—Revised. J. Foot Ankle Res..

[46] Woodburn J., Barker S., Helliwell P.S., Woodburn J. (2002). A randomized controlled trial of foot orthoses in rheumatoid arthritis. J. Rheumatol..

[47] Van der Leeden M., Steultjens M., Dekker J.H.M., Prins A.P.A., Dekker J. (2006). Forefoot joint damage, pain and disability in rheumatoid arthritis patients with foot complaints: The role of plantar pressure and gait characteristics. Rheumatology.

[48] Gaino J.Z., Bértolo M.B., Nunes C.S., de Barbosa C.M., Landim S.F., Sachetto Z., Magalhães E.d.P. (2021). The effect of foot orthoses on balance, foot function, and mobility in rheumatoid arthritis: A randomized controlled clinical trial. Clin. Rehabil..

[49] De Mits S., Mielants H., De Clercq D., Woodburn J., Roosen P., Elewaut D. (2012). Quantitative assessment of foot structure in rheumatoid arthritis by a foot digitizer: Detection of deformities even in the absence of erosions. Arthritis Care Res..

[50] Rome K., Clark H., Gray J., McMeekin P., Plant M., Dixon J. (2017). Clinical effectiveness and cost-effectiveness of foot orthoses for people with established rheumatoid arthritis: An exploratory clinical trial. Scand. J. Rheumatol..

[51] Bagherzadeh Cham M., Ghasemi M.S., Forogh B., Sanjari M.A., Zabihi Yeganeh M., Eshraghi A. (2014). Effect of rocker shoes on pain, disability and activity limitation in patients with rheumatoid arthritis. Prosthet. Orthot. Int..

[52] Barn R., Brandon M., Rafferty D., Sturrock R.D., Steultjens M., Turner D.E., Woodburn J. (2014). Kinematic, kinetic and electromyographic response to customized foot orthoses in patients with tibialis posterior tenosynovitis, pes plano valgus and rheumatoid arthritis. Rheumatology.

[53] González-Fernández M.L., Valor L., Morales-Lozano R., Hernández-Flórez D., López-Longo F.J., Martínez D., González C.M., Monteagudo I., Martínez-Barrio J., Garrido J. (2016). To what extent is foot pain related to biomechanical changes and ultrasound-detected abnormalities in rheumatoid arthritis?. Clin. Exp. Rheumatol..

[54] Stewart S., Dalbeth N., Aiyer A., Rome K. (2020). Objectively Assessed Foot and Ankle Characteristics in Patients With Systemic Lupus Erythematosus: A Comparison with Age- and Sex-Matched Controls. Arthritis Care Res..

[55] Bal A., Aydog E., Aydog S.T., Cakci A. (2006). Foot deformities in rheumatoid arthritis and relevance of foot function index. Clin. Rheumatol..

[56] Göksel Karatepe A., GüNaydin R., Adibelli Z.H., Kaya T., DuruöZ E. (2010). Foot deformities in patients with rheumatoid arthritis: The relationship with foot functions. Int. J. Rheum. Dis..

[57] Biscontini D., Bartoloni Bocci E., Gerli R. (2009). Analysis of Foot Structural Damage in Rheumatoid Arthritis: Clinical Evaluation by Validated Measures and Serological Correlations. Reumatismo.

[58] Tehan P.E., Morpeth T., Williams A.E., Dalbeth N., Rome K. (2019). «come and live with my feet and you’ll understand»—A qualitative study exploring the experiences of retail footwear in women with rheumatoid arthritis. J. Foot Ankle Res..

